# Gestational Weight Gain: Is the Role of Genetic Variants a Determinant? A Review

**DOI:** 10.3390/ijms25053039

**Published:** 2024-03-06

**Authors:** Reyna Sámano, Hugo Martínez-Rojano, Gabriela Chico-Barba, Ricardo Gamboa, María Eugenia Mendoza-Flores, Francisco Javier Robles-Alarcón, Itzel Pérez-Martínez, Irma Eloisa Monroy-Muñoz

**Affiliations:** 1 Coordinación de Nutrición y Bioprogramación, Instituto Nacional de Perinatología, Secretaría de Salud, Mexico City 11000, Mexico; gabyc3@gmail.com (G.C.-B.); tina14mx@yahoo.com (M.E.M.-F.); 2 Programa de Posgrado Doctorado en Ciencias Biológicas y de la Salud, División de Ciencias Biológicas y de la Salud, Universidad Autónoma Metropolitana, Mexico City 04960, Mexico; 3 Sección de Posgrado e Investigación de la Escuela Superior de Medicina del Instituto Politécnico Nacional, Mexico City 11340, Mexico; hmartinez_59@yahoo.com.mx; 4 Departamento de Fisiología, Instituto Nacional de Cardiología “Ignacio Chávez”, Mexico City 14080, Mexico; rgamboaa_2000@yahoo.com; 5 Facultad de Nutrición, Universidad Autónoma del Estado de Morelos, Cuernavaca 62350, Mexico; francisco.robles@uaem.edu.mx (F.J.R.-A.); itzel.perez@uaem.edu.mx (I.P.-M.); 6 Departamento de Investigación Clínica en Salud Reproductiva y Perinatal, Instituto Nacional de Perinatología, Secretaría de Salud, Mexico City 11000, Mexico

**Keywords:** gestational weight gain, cytokines, pregnancy, BMI, genetic variants

## Abstract

Excessive or insufficient gestational weight gain (GWG) leads to diverse adverse maternal and neonatal outcomes. There is evidence that pregestational body mass index (pBMI) plays a role in GWG, but no genetic cause has been identified. In this review, we aim to analyze genotype variants associated with GWG. Results: We identified seven genotype variants that may be involved in GWG regulation that were analyzed in studies carried out in Brazil, Romania, the USA, Turkey, Ukraine, and Canada. Some genetic variants were only associated with GWG in certain races or depending on the pBMI. In women who were obese or overweight before gestation, some genetic variants were associated with GWG. Environmental and genetic factors together showed a greater association with GWG than genetic factors alone; for example, type of diet was observed to have a significant influence. Conclusions: We found little scientific evidence of an association between genotype variants in countries with a high prevalence of women of reproductive age who are overweight and obese, such as in Latin America. GWG may be more dependent on environmental factors than genetic variants. We suggest a deeper study of genetic variants, cytokines, and their possible association with GWG, always with the respective control of potential cofounding factors, such as pBMI, diet, and race.

## 1. Introduction

Pregnancy results in changes to maternal metabolism, which can be influenced by environmental, genetic [1], and inflammatory factors [2,3]; the combination of these factors could result in altered gestational weight gain (GWG) [3] and/or an increased risk of developing gestational diabetes [4], preeclampsia [5], and pregnancy-induced hypertension [6], among others.

Worldwide, excessive or inadequate GWG affects up to 70% of pregnancies [7,8], with excessive GWG being more frequent, accounting for 35 to 60% [8,9]. Excessive GWG increases the risk of morbidity and the costs for those who experience these adverse effects [10]. Pregestational obesity and overweight are factors associated with high odds of maternal and neonatal morbidity, even mortality [11]. Nevertheless, some genetic variants [12] interact with cytokines and adipokines [2,13] to modify pregnancy homeostasis, including GWG [14].

Maternal health during pregnancy may be affected by the interaction and mutual influence between genetic and environmental factors such as food intake, sleep quality, and physical activity. Increased knowledge of the role of how GWG increases the risk of adverse pregnancy outcomes is essential to reduce these risks in the future, as well as to understand how these risk factors may vary depending on pregestational BMI (pBMI) [13,15,16] or race, as was reported by Groth et al. for Caucasian and African American women [17]. The role of genetic variants with respect to GWG is uncertain. Nevertheless, overweight and obesity are increasing in low- and middle-income countries. We aimed to analyze genotype variants associated with GWG reported in the scientific literature to understand their roles in gestational weight gain.

## 2. Relevant Sections

### 2.1. What Is the Role of Genomic Sciences in Body Weight?

Genomic sciences allow for a better understanding of the role energy from food intake in the human body plays via signaling pathways that could explain the accumulation of normal and abnormal body fat. Genome-wide association studies contribute by facilitating the identification of genes present in all chromosomes. Single-nucleotide variants (SNV), formerly known as single-nucleotide polymorphisms (SNPs), are classified as functionals (rSNP and srSNP) and encoders (cSNP). rSNPs are located in the promoter sequence, and rsSNPs can be located in introns or in the codifying sequence [18]. Specific functional genetic variants are recognized as being genetic markers for diabetes, hypertension, and development. Genetic expression depends on cis regulators, such as the promoter sequence, and trans regulators, like transcriptional factors. The latter can join DNA, RNA polymerase II, and other molecules that modulate the genetic expression of specific tissues like fat or muscle [19].

Single-nucleotide variants (SNVs) are the result of replacing one nucleotide with another and could result in a structural and biological function change in the protein encoded by the gene in question. An SNV in the promoter region of a gene can increase or decrease the production of relevant proteins [20]. These inherited single-nucleotide variants could be associated with a higher or lower risk of developing a particular disease because SNVs can affect proteins or protein derivatives, such as neurotransmitters, protein-related obesity, and protein receptor leptin, among others. Therefore, their function can be altered.

#### 2.1.1. Which Geographical Areas Are Studied, and What Are the Principal Genetic Variants Associated with Gestational Weight Gain?

The association between specific genetic variants and excessive GWG has been evaluated in 6974 women. The most recent studies came from the United States of America [17,21,22,23,24], Brazil [25,26,27,28], Romania [29,30], Turkey [31,32], Ukraine [33,34], Canada [35], Germany [36], China [37], and Poland [38]. These studies were performed on pregnant adult women (aged 22–35). As can be observed, only three countries from America, five from Europe, and one from Asia analyzed the probable association between genetic variants and gestational weight gain. Whether excessive or insufficient GWG, the two conditions have high rates, with 14–33% being insufficient and 26–61% being excessive [7,8]. This association is not fully addressed in regions like Latin America, characterized by countries with a high prevalence of overweight and obesity in reproductive-age women like Mexico. Table 1 shows the characteristics of the papers included in this review, further developed in the following text.

#### 2.1.2. Genotype Variants Associated with GWG

Of all genetic variants in this manuscript, some are associated with GWG, while others are only associated with pregestational BMI. One of these genetic variants is located in *LEPR* (leptin receptor); this gene codes for the leptin receptor (an adipocyte-specific hormone that regulates body weight) and controls fat metabolism and other essential functions. Changes in this gene have been associated with obesity and pituitary dysfunction [39,40]. In a sample of 147 adult women aged 27, it was observed that *LEPR* rs7799039 GG carriers had a lower body weight throughout pregnancy than AA or GA carriers. The authors concluded that the rs7799039 genotype was associated with a high risk of developing excessive GWG [25]. As explained, 98% of placental secreted leptin enters circulation, and the placenta and adipose tissue express this gene during pregnancy [41]. In other studies, the AA genotype was more associated with greater amounts of adipose tissue in obese people than the GG genotype, but this association is not present in other tissues [42,43].

Another studied gene is the fat mass and obesity-associated (*FTO*) gene, which controls energy balance [44]. It codes for the 2-oxoglutarate-dependent nucleic acid demethylase and is expressed in the hypothalamus. Female carriers of the AT *FTO* rs9939609 genotype have a greater risk of excessive GWG than those with other genotypes, but only during the early stages of pregnancy. In females with type 1 or 2 diabetes, this genotype is associated with an increased risk of excessive GWG, as reported in a study performed on a group of 70 Brazilian adults with type 1 or 2 diabetes mellitus [26]. Among women who self-identify as black with pregestational obesity, the *FTO* rs99339609 genetic variant showed an association with GWG. In 194 African American women, the AA *FTO* rs 9939609 genotype was associated with higher GWG compared to the TT genotype. The AA and AT genotype carriers gained 7.6 kg and 3.6 kg more than the TT carriers, respectively [17].

Similarly, in 97 low-income black women, the AA genotype was associated with greater GWG (16.4 kg) compared with the AT and TT genotypes (14.0 kg and 12.2 kg, respectively) [21]. Nevertheless, for Caucasian women, the results were unexpected because they did not show any associations; only African American women with obesity gained 6.7 kg more than those with TT or AT genotypes [17]. Race may play a role in the expression of the genetic variants; this may, however, vary depending on pBMI, and thus further studies, with larger sample sizes, are necessary to clarify this issue.

The monoamine oxidase A (*MAO-A*) gene has also been associated with GWG. *MAO-A* is a member of a family that encodes mitochondrial enzymes, which catalyze the oxidative deamination of amines such as dopamine, norepinephrine, and serotonin. *MAO-A* maintains neurotransmitter metabolism and hence influences appetite and food intake. Only one study performed on 93 Canadian female carriers of the *MAO-A* 4/4 genotype observed a higher frequency of excessive GWG than 3/4 and 3/3 carriers; the possible explanation for this is reduced activity of dopamine caused by its deamination. The presence of *MAO-A* 4/4 (with high activity) in women was associated with excessive GWG when compared with 3/3, 3/4, or 3/3.5 (19.3 ± 4.1 vs. 17 ± 5 kg) [35]. The dopamine pathway could be the cause of this phenomenon.

Other studies explored the association of the *GNB3* gene with GWG. In 2018, Groth and Cols. Ref. [17] reported that African American women with pregestational obesity and the CC *GNB3* rs5443 genotype were associated with greater GWG (kg) than TT and CT carriers. Nevertheless, Caucasians did not exhibit an association with GWG.

On the other hand, the peroxisome proliferator-activated γ-receptor (*PPAR*γ) rs1801282 SNV and its relationship with GWG were analyzed in 97 Ukrainian [34] and 162 Turkish women [32]; the authors concluded that based on adjusted models, Ala carriers had a greater probability of undergoing excessive GWG. In addition, the TT genotype from endothelial nitric oxide synthase (eNOS) *Glu298Asp* SNV in the Ukrainian sample was related to an increment of 5 kg compared to the other genotypes (GT and GG) [33].

#### 2.1.3. Are Foods Important, in Addition to Genetic Variants?

It is possible that, as a consequence of the decontrolling of serum lipids and other substances, GWG is affected by genetic variants. Regarding the *Glu298Asp* (G894T) SNV of the *eNOS* gene (TT), in 97 adult women during the first and third trimester of pregnancy, TT was associated with a 1.5-fold increment in GWG compared to women with the GG genotype. The adequate, insufficient, and excessive amounts of GWG were 34, 19.6, and 46.4%, respectively [33].

Like others, the potassium channel tetramerization domain containing 15 (*KCTD15*) gene has been associated with the selection and intake of food providing high amounts of energy and with high carbohydrate and fat content [45]. Like other obesity-related genes, it is highly expressed in the hypothalamus [46]. In KCTD15 rs11084753 AG carriers, an association with GWG was observed, resulting in a gain of 16.9 kg vs. 12.3 for the GG genotype (*p* = 0.018) and vs. 10.9 kg for AA carriers (*p* = 0.026). Women carriers of *KCTD15* rs11084753 gained more weight during pregnancy, highlighting that this occurred when the women consumed more fatty foods than those with other genotypes [22]. The probable explanation is that fat intake could modify the effect of the *KCTD15* rs11084753 genetic variant gene on GWG [23]. Then, the environment could modify the effect of the SNV, which is demonstrated by an attenuated effect on body weight in *FTO* rs9939609 carriers that maintain a healthy lifestyle [47] and an optimal food intake [48]. Parallel findings were reported by Lawlor in adult UK women [49] and in African American women: diet plus SNV presence explained the observed GWG [22], with only 9% variability [23]. Genetic variants are intrinsic factors that pose a risk of developing excessive GWG. However, maintaining a healthy lifestyle and nutrition during pregnancy could reduce this risk and lead to a healthy childhood and adulthood [44].

Additionally, the effect of different types of diet on GWG in the presence of *FTO* gene (rs9939609, rs17817449) and *ADRB2* (rs1042713, rs1042714) variants has been addressed in Brazilian pregnant women with pregestational diabetes. This nutrigenetic trial randomly assigned 70 pregnant women to a traditional diet (*n* = 41) or DASH diet (*n* = 29). Santos et al. found that regardless of type of diet, AT carriers of *FTO* rs9939609 and AA carriers of *ADRB2* rs1042713 had higher risk of earlier excessive GWG [26].

To date these are the only studies where the effect of nutritional factors has been evaluated on GWG in the presence of genetic variants; further investigation must be addressed to clarify the impact diet has on weight gain.

#### 2.1.4. Are Genetic Variants More Associated with Pregestational BMI Than with GWG?

Some genetic variants are associated with GWG, but others are not. For example, in Brazilian women, *FTO* rs17817494 was not associated with GWG, while women carriers of AT, AA, and TT gained 13.0 kg, 13.2 kg, and 12.3 kg, respectively (*p* = 0.15); the low sample size could explain this finding. This finding corresponds to 93 Caucasian women from Canada [35].

In Brazilian pregnant women, the *LEPR* rs1137101 genetic variant was associated with pregestational overweight or obesity. Hence, this alteration can be expressed before pregnancy, but the effects could be different for women carriers and their offspring during gestation [25]. For example, in another SNV, the *FTO* rs17817449, women carriers with different diets exhibited no associations with GWG. Goldfield et al. demonstrated that there was a null association between *DAT-1* gene variants and GWG [35].

Information with more consistency shows that *FTO* genetic variants are associated with pregestational status, as shown in a study carried out on 205 Turkish adult women; this study reported that the *FTO* AA genotype was associated with a risk of pre-pregnancy overweight/obesity (OR = 1.43, 95% CI [1.25–3.4], *p* = 0.035). The authors concluded that the AA *FTO* rs9939609 genotype was not associated with excessive GWG after adjusting for pre-pregnancy weight (*p* > 0.05). It seems that *FTO* rs9939609 affects pregestational weight and GDM, an effect that could be explained by the insulin resistance pathway [31]. In the same way, *FTO* rs9939609 could have different effects or expression in diverse races. In a study on 406 Brazilian adult women, it was reported that there was a higher frequency of excessive GWG in those with the TT genotype (51%) compared to those with other genotypes; however, this finding did not have statistical significance, and Caucasian women did not report any associations. Only this genetic variant was associated with pBMI and the period after delivery [27] until 2–4 years postpartum. This phenomenon could begin to occur at young ages via the LEPR SNV, which could modify some metabolic indicators [50]. The *MAOA* genetic variant is associated with BMI in female adolescents [51] due to an effect deriving from stressor factors.

Another relevant gene is the melanocortin-4-receptor (*MC4R*). The protein encoded by this gene is a membrane-bound receptor and a member of the melanocortin receptor family. Defects in this gene and other heterogeneous factors cause autosomal dominant obesity [52]. The MC4R is located in the brain. It has an affinity with melanocyte alpha, beta hormone stimulators, and melanocortin and corticotropin hormones. Regarding GWG, in a sample of 185 Romanian pregnant women, *MC4R* rs17782313 showed no associations with GWG, only with pBMI [29]. Lawlor et al. reported parallel findings for adult women from the UK [49].

Melanocortin 4 receptor (*MC4R*) deficiency due to the disruption of one or both MC4R alleles has a relationship with the frequency of obesity [44]. Nevertheless, the role of the genetic variant *GNB3* rs5443 (guanine nucleotide-binding protein rs5443) in GWG is uncertain, as was observed in a study incorporating women from the USA. Of the 674 African American and Caucasian women who participated in the study, in the African American group of women with pregestational overweight or obesity, those with TT or CT genotypes gained 7.3 and 6.0 kg, respectively, more than women with CC genotypes. Caucasian women did not show any association of *GNB3* rs5443 with GWG [17]. However, in a sample of 158 Romanian pregnant adults, there was a marginally positive association between the variant genotype *GNB3* rs5443 and excessive GWG [30]. Hence, more research is needed to confirm these findings. In this study, pBMI was a better predictor of GWG than the *GNB3* SNV.

It is possible that pregestational diabetes type 1 or 2 is associated with previous BMI and that this, in turn, is associated with GWG, but there is not enough scientific evidence; for example, in a case–control association study on the SNV of peroxisome proliferator-activator receptor-gamma2 (*PPAR-γ2*) carried out in Turkey, women with gestational mellitus diabetes had greater gestational weight gain than women without diabetes. The conclusion was that *PPAR-γ2* could influence glucose metabolism and pBMI [32]. This hypothesis has been inconsistent with other studies. The effect of this SNV could be affected by race; for example, in 97 Caucasian Ukrainian women, the genetic variant of *PPAR-γ* Pro12Ala was associated with greater GWG and a high frequency of excessive GWG [34].

Previous reports complement the following finding. The adrenoceptor beta 2 (*ADRB2*) gene predisposes one to a high probability of developing obesity and is expressed in adipose tissue and during lipolysis [53]. To give another example, 70 Brazilian women with type 1 or 2 diabetes mellitus and who also carried the AA genotype of *ADRB2* rs1042713 had a greater risk of high gestational weight gain than GG carriers (aHR 3.91; CI 95% 1.12–13.70; *p* = 0.03) during the early stages of pregnancy, regardless of their diet [26]. Even *MAOA* genetic variants have been associated with BMI in female adolescents [51].

As previously mentioned, the *eNOS* TT single-nucleotide variant is related to greater levels of triglycerides, cholesterol and low-density lipoprotein, glucose, and insulin and with greater HOMA-IR scores when compared with the GT genotype during the third trimester due to decreased activity of *eNOS*, particularly among those with excessive GWG [33]. It is possible that the activity of the *PPAR-γ* gene toward lipids and carbohydrate metabolism could have a positive association with GWG [34].

Several polyunsaturated fatty acids can be modified and affect adipocyte storage. The *FADS2* gene codes for a desaturase enzyme that interacts in the process of endogenously converting 18-carbon polyunsaturated fatty acids into very-long-chain fatty acids [36]. Some single-nucleotide variants in these genes are related to the bioavailability of polyunsaturated fatty acids [36,37,38,39,40,41,42,43,44,45,46,47,48,49,50,51,52,53,54]. In a sample of 185 Brazilian adult women, the GG *FADS2* rs174575 genotype was associated positively with gestational weight gain (coefficient 0.11; *p* = 0.016; 95% CI 0.10–0.19) [28]. The alteration in genetic transcription probably reduces the bioavailability of ω-3 and promotes the storage of ω-6 and its derivatives [28]. On the other hand, the association between *GCKR* rs780094 (TT reference, T > C risk) and GWG has been studied. Nevertheless, the results were different. In 158 Romanian adult women, the *GCKR* (glucokinase receptor) rs 780094 genetic variant was not associated with GWG. As can be seen, the effect of the SNVs is not the same in all races. This gene codes for a protein that inhibits glucokinase in liver and pancreatic islet cells by binding non-covalently to form an inactive complex with the enzyme [30].

### 2.2. Genetic Variants and Cytokines and Their Probable Interaction with Gestational Weight Gain

Mature adipocytes and a stromal vascular constitute adipose tissue; they function as a unit regulating multiple endocrine and metabolic activities through adipokines [55,56]. It has been shown that the adipose tissue of obese subjects releases mainly proinflammatory adipokines, of which the most important are leptin, visfatin, resistin, TNF-α, IL-6, angiotensin II, and plasminogen activator inhibitor 1. Anti-inflammatory cytokines, such as TGF-β, adiponectin, IL-10, IL-4, IL-13, IL-1 receptor antagonist (IL-1Ra), and adipolin, are secreted by lean subjects [57].

Leptin was the first adipokine to be discovered [56]. *LEPR* rs7799039 is a nucleotide variant located in the promoter region of the *LEPR* gene, and it has been associated with a higher risk of GWG and type 2 diabetes mellitus development. *LEPR* rs1137101 (Q223R), is located in the region that codes for the extracellular domain of the leptin receptor; therefore, the amino acid change affects all isoforms of the receptor, resulting in an amino acid variation in the extracellular region of the leptin receptor, which can change the functional characteristics of the receptor [58,59]. Both variants reduce the function of LEPR, leading to a higher risk of obesity development. It is known that leptin and its receptor can induce IL-6 and TNF-α gene expression, so any change in *LEPR* function could lead to an altered expression of these two cytokines.

It has been reported that there is a possible association between the *FTO* gene and leptin. *FTO* rs9939609 and rs17817449 are single-nucleotide variants located in the intronic region of the *FTO* gene. *FTO* rs9939609 has been associated with leptin gene expression [60]. The *FTO* rs178117119 is implicated in the BMI-mediated risk of developing type 2 diabetes mellitus among obese children and adolescents. Therefore, if *FTO* can regulate *LEP* and *LEPR* expression, this could also lead to an altered expression of IL-6 and TNF-α and a significant susceptibility to the development of chronic diseases such as type 2 diabetes mellitus.

*KCTD15*, during embryogenesis, inhibits binding protein 2 (AP-2) transcriptional activities via interaction with its activation domain. The probable molecular basis of *KCTD15* in obesity may involve some important factors in adipogenesis regulated by *AP-2* [61]. The higher risk of GWG precipitated by rs11084753 *KCTD15* could lead to the dysregulation of *AP-2* [62]. In skin cultures designed to mimic cutaneous inflammation and wound healing, it has been demonstrated that the AP-2 protein is functionally important in TGF alpha-induced VPF/VEGF gene expression. Consequently, as obesity induces an inflammatory state, the mechanism could be similar to the one observed in skin diseases and the healing process [63].

*MAO-A*’s role consists of generating *ROS* (H2O2) in alternatively activated monocytes/macrophages. Interesting studies have shown that MAO-produced *H2O2* is an essential inhibitor of inducible nitric oxide synthase (*NOS2*) expression. Although NOS2 generation of NO is differently regulated in murine and human macrophages, it has been speculated that the expression of *MAO-A* in anti-inflammatory macrophages could partially stabilize this phenotype by preventing the expression of *NOS2* and the generation of NO [64]. Excessive amounts of adipose tissue (specifically visceral) generate high quantities of reactive oxygen species (ROS) associated with obesity-related pathologies. Nevertheless, *MAO-A*’s role in obesity development has not been fully elucidated. The association of *MAO-A* variants with GWG constitutes a practical initial approach [65].

Variable number tandem repeat (VNTR) variants have been described in the 3′ region of dopamine transporter *(DAT-1)*, with 9-repeat and 10-repeat forms being the most common. The 9-repeat allele could induce lower levels of postsynaptic dopamine. As dopamine regulates food intake, it has been proposed that genetic variants found in dopamine-associated molecules, like *DAT-1*, could indirectly influence weight gain. However, this association has not yet been verified. This could be due to the modulating effect of other proteins like MAO. It is well understood that *MAO* is involved in degrading biological amines, including dopamine, and thereby deaminates dopamine that has been taken back up by *DAT-1* [64,66].

Another regulatory factor of nutrient metabolism is *PPARγ*; it controls cellular lipid metabolism with anti-inflammatory activities. *PPARγ* rs1801282 is a missense mutation resulting in an amino acid substitution of alanine for proline at codon 12 (Pro12Ala); this variant is located in the ligand-independent activation domain that reduces *PPARγ* transcriptional activity. In a study involving pregnant women and their offspring in Spain, no statistically significant differences were identified in TNF-α, IL-6, and IL-10 cytokine levels at any time point in pregnancy between CG carriers and those carrying the CC genotype, and no effect was observed on their offspring [67].

Regarding *GNB3* rs5443, it is located on exon 10 of *GNB3* and is associated with the generation of a splice variant named Gβ3. This variant is characterized by a deletion of 41 amino acids and one WD repeat domain in the G beta subunit; both characteristics seem to enhance its function with respect to signal transduction, including lipolysis [64]. *GNB3* rs5443 has also been associated with chemotaxis, antigen-specific cellular immune response, and IL-2-induced proliferation [68].

Finally, in an in vitro study, the effect of *eNOS* Glu298Asp on cytokine production was evaluated, demonstrating increased levels of inflammatory cytokines (IL-1, IL-6, and TNF-α) in the TT genotype. This could explain why people with the T allele present chronic inflammation and/or do not respond to anti-inflammatory treatments or exhibit inflammation and obesity [69].

**Figure 1:**
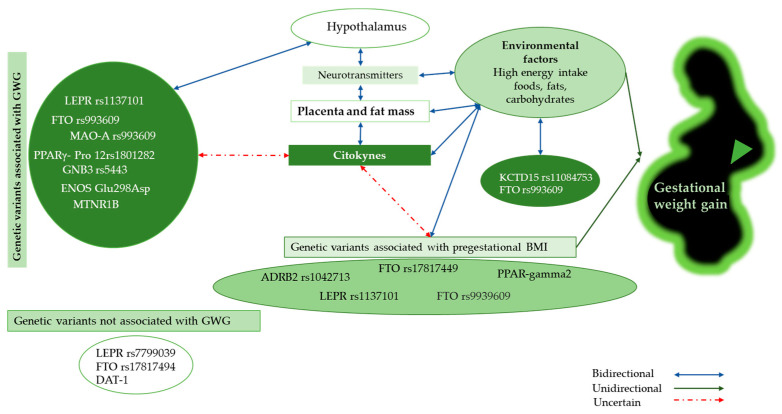
Interaction between genotype variants and other factors associated with GWG. Abbreviations. GWG: Gestational weight gain, BMI: Body mass index, LEPR: Leptin receptor, FTO: Fat mass and obesity-associated, GNB3: Guanine nucleotide-binding protein 3, MAO-A: monoamine oxidase A, PPARγ: Peroxisome proliferator-activated γ-receptors, eNOS: Endothelial nitric oxide synthase, MTNR1B: Melatonin receptor 1B, KCTD15: Potassium channel tetramerization domain containing 15, ADRB2: Adrenoceptor beta2, and DAT-1: dopamine transporter.

Figure 1 shows the genetic variants associated and unassociated with GWG; here, we identify that some genetic variants are associated with pBMI but not with GWG. Among neurotransmitters, serotonin, dopamine, and norepinephrine were highlighted. Nevertheless, the role of cytokines (leptin, visfatin, resistin, TNF-α, IL-6, angiotensin II, plasminogen activator inhibitor 1, TGF-β, adiponectin, IL-10, IL-4, IL-13, IL-1 receptor antagonist (IL-1Ra), and adipolin) is not clear. In our analysis, environment was a determinant in the effect of genetic variants on GWG.

## 3. Discussion

Previous studies concluded that some genetic variants could be associated with GWG. However, indirectly, as reported by Stuebe [24], genetic variants associated with pregestational weight have some mediators that affect serum lipids and fat storage and are a cause of diabetes [28,29,36,37,38,39,40,41,42,43,44,45,46,47,48,49,50,51,52,53,54]. Accordingly, this process can be supported by genes related to GWG. Considering their effect on pBMI, they could play a role in insulin signaling, glucose homeostasis, mitochondrial metabolism, and inflammatory responses, at least in women with type 1 diabetes mellitus [38].

Genetic variants can affect GWG, cytokine production, and the birth weight of offspring. In 1114 mother–neonate dyads, *MTNR1B* was associated with GWG and the offspring’s birth weight. The authors of the associated study proposed that offspring could have a high risk of obesity during childhood [70]. Likewise, a study involving 1025 Chinese women reported that GWG could affect the circadian-cycle-related *MTNR1B* rs10830963 genetic variant, with severe repercussions on long-term glycemic changes. Thus, as can be seen, the effect of GWG transcends pregnancy [71] in women with a history of DMG.

Nevertheless, generating more enlightening insights into the gene–diet–environmental interaction regarding GWG is necessary. The environment determines the phenotype generated by the SNVs’ presence, as revealed in a study involving 1153 elderly Swedish men. They were followed for 32 years; they were born between 1920 and 1924, and the *FTO* rs9939609 variants did not reveal any difference in BMI. They were born before ultra-processed foods and fast foods were widely available and before physical activity levels decreased. Thus, food and physical activity could influence the effect of the presence of *FTO* rs9939609 [72]. It should be highlighted that the previous study was performed only in older men and a different scenario could be present in women at reproductive age. Therefore, these findings need to be interpreted with caution.

### 3.1. Limitations and Strengths

Our review has several limitations. The first is that for the group of pregnant women, there is no information about the interaction between genetic variants, cytokines, and GWG in the same study. Therefore, using the evidence reported in other age groups of non-pregnant individuals regarding their association with cytokines, we hypothesize that cytokines could have similar effects in some molecules and GWG, but in others, these effects could be different; this line of research needs to be scrutinized and developed during pregnancy. Our review’s strength is that it is the first to explore the genotype variants associated with GWG. We observed that low- and middle-income countries have little or no research on this topic, yet they have high rates of overweight and obesity in their female populations.

### 3.2. Clinical Implications

Pregnancy is an inflammatory state per se that could be dysregulated by inflammatory conditions like obesity. The genotypification of variants of GWG-related genes during pregnancy not only allows for a better understanding of the balance needed between food intake and weight gain during this stage but could also provide a risk probability for developing overweight or obesity and consequently the risk of developing complex diseases, like type 2 diabetes mellitus, which, in most cases, lead to severe consequences for maternal and child health. It also highlights the need to control the intake of high-calorie foods and maintain routine physical activity to avoid excessive GWG.

## 4. Conclusions

It has been found that some genetic variants are associated with GWG. Nevertheless, this association is only significant for African American women with overweight or obesity. Due to a lack of information, the role of cytokines in the association between genetic variants and GWG remains to be determined.

## 5. Future Directions

Further research is needed to understand the probable explanation for the association between genetic variants, environmental factors such as high-energy food intake, and their interaction with excessive or inadequate GWG in some ethnic groups, like those from low- and middle-income countries.

## Figures and Tables

**Table 1:** Analysis of the information on genetic variants in women with GWG.

Author/Year/Country	Sample (n and Phenotype)	Design	Study Period	Genetic Variant (Exposure)	Gestational Weight Gain (kg)	Findings
Martins [25]/2018/Brazil	147 Age range: 20–40 years old	Prospective cohort	From gw 5 to delivery	LEPR rs1137101 (blood) real-time PCR	AA: 12.1 AG: 13.2 GG: 11.7	No association of AG + GG vs. AA with GWG. RR 1.63 (95% CI 0.78–3.42) AM
Martins [25]/2018/Brazil	147 Age range: 20–40 years old	Prospective cohort	From gw 5 to delivery	LEPR rs7799039 (blood) real-time PCR	AA: 12.8 AG: 11.7 GG: 13.2	Association of GA + AA vs. GG with excessive GWG. RR: 2.18 (95% CI 1.27–3.71) AM
Groth et al. [21]/2015/USA	97 Black women, low-income Age range: 18–36 years old	Prospective cohort	From gw 20 to 6 months postpartum	FTO rs993609 (buccal epithelial cells) real-time PCR	AA: 16.4 AT: 14.0 TT: 12.2	Association with greater GWG in AA and AT vs. TT. TT gene predicted GWG (b = −13.48, *p* = 0.007) AM
Groth et al. [17]/2018/ USA	770 580 Caucasian 194 African American Age range: 12–35 years old	Prospective cohort	From gw 20 to 1 year postpartum	FTO rs993609 (buccal epithelial cells) real-time PCR	Obese African American had greater GWG AA: 7.6 AT: 3.6	Obese African American women homozygous for the AA genotype had higher GWG vs. TT genotype (*p* = 0.006).
Meng Y et al. [23]/2018/USA	55 women. Age range: 18–36 years old	Prospective cohort	From gw 20 to 6 months postpartum	GNB3, FTO, MC4R, KCTD15, NEGR1, SH2B1, and GNPDA2 (buccal epithelial cells) real-time PCR. Only KCTD15 rs11084753 showed association	KCTD15 rs11084753 GG: 12.3 GA: 16.9 AA: 10.9	Gene risk score explained 9.1 of variability in GWG. Values for GA (mean: 16.9 kg; 95% CI: 14.5 to 19.4 kg), GG (mean: 12.3 kg; 95% CI: 10.2 to 14.5 kg; *p* = 0.018); and AA carriers (mean: 10.9 kg; 95% CI: 7.3 to 15.0 kg; and *p* = 0.026) were obtained when the individuals consumed more fat than those with other genotypes.
Goldfield [35]/2013/Canada	93 Caucasian women. Age range: 27.5–35.9 years old	Prospective cohort	From gw 14–20 to delivery	DAT-1 gene (blood) determined via end-point PCR	10/10 genotype 17.5 ± 4.7 9/9 genotype 17.5 ± 4.9	No association of DAT-1 variants with excessive GWG: 10/10 genotype vs. 9/9 genotype (17.5 ± 4.7 vs. 17.5 ± 4.9, *p* = 0.98)
Goldfield [35]/2013/Canada	93 Caucasian women. Age range: 27.5–35.9 years old	Prospective cohort	From gw 14–20 to delivery	MAO-A (blood) determined via end-point PCR	4/4 genotype 19.3 ± 4.1 3/3, 3/4, or 3/3.5 pooled genotypes 17.0 ± 5.0	Association of 4/4 genotype with greater GWG: 4/4 vs. 3/3,3/4,3/3.5 19.3 ± 4.1 kg versus 17.0 ± 5.0, *p* = 0.03
Groth et al. [17]/2018/ USA	770 580 Caucasian 194 African American Age range: 12–35 years old	Prospective cohort	From gw 20 to 1 year postpartum	GNB3 rs5443 (buccal epithelial cells) determined via real-time PCR	African American with obesity CT genotype −6.6 vs. TT genotype	Greater association of GNB3 CT genotype with decreased GWG compared to those with the TT genotype (−6.6 kg., *p* = 0.011) in obese African American
Ostafiichuk et al. [34]/2018/Ukraine	97 Ukrainian women with a normal pregestational BMI. Age range: 24–30 years old	Prospective cohort	From 10 gw to delivery	PPAR- γ Pro 12 rs1801282 (Blood) real-time PCR	Homozygous Pro/pro 12.1 ± 2.6 (CI 11.1–13.1) Ala carriers: 19.5 ± 2.7 (CI 17.8–21.2)	Association of Ala carriers with increased GWG. GWG is 1.6 times higher in Ala carriers compared to the Pro/Pro genotype (*p* < 0.05)
Ostafiichuk et al. [33]/2022/Ukraine	97 Ukrainian women with a normal pregestational BMI. Age range: 24–30 years old	Prospective cohort	From gw 9 to 38–40	eNOS Glu298Asp (Blood) real-time PCR	GG: 12.5 ± 2.5 (95%CI 11.3–13.4) GT: 14.4 ± 3.1 (95%CI 13.1–15.7) TT 19.3 ± 2.3 (95%CI)	Association of TT with GWG. GWG is 1.5 times higher in women with TT than in GG carriers (OR = 4.52; 95%CI: 1.18–17.32; *p* < 0.05)
Tok et al. [32]/2006/Turkey	162 Caucasian women. Cases: 62 patients with gestational diabetes. Age range: 28.4–38.2 Years old Control: 100 patients with non-gestational diabetes. Age range: 27.1–38.5 years old	Cases and controls	From 28 to delivery	PPAR- γ Pro 12 rs1801282 (Blood) real-time PCR	Control Pro12Ala: 10.8 Pro12Pro: 9.9, *p* = 0.64 Case Pro12Ala: 12.2 Pro12Pro: 17.9 *p* = 0.017	Women with GDM and Pro12 Ala from PPAR-gamma2 were associated with GWG. AM: 17.9 ± 13.9, *p* = 0.017

gw: gestational week. AM: Adjusted models. PCR: Polymerase chain reaction. GWG: Gestational weight gain. aHE: Adjusted hazard ratio RCT: randomized clinical trial. CI: confidence interval. GDM: Gestational Diabetes Mellitus.

## Data Availability

All data generated or analyzed during this study are included in this published article.

## Abbreviations

GWG: Gestational weight gain; BMI: Body mass index; MeSH: Medical subject headings; IOM: Institute of Medicine; pBMI: Pre-pregnancy body mass index; CI: Confidence interval, gw: Gestational week; AM: Adjusted models; PCR: Polymerase chain reaction; aHE: Adjusted hazard ratio; VNTR: Variable number tandem repeat; ROS: Reactive oxygen species; no: Nitric oxide; GDM: Gestational diabetes mellitus; SNV: Single-nucleotide variant; *LEPR:* Leptin receptor gene; LEPR: Leptin receptor, FTO: Fat mass and obesity-associated, GNB3: Guanine nucleotide-binding protein 3, MAO-A: monoamine oxidase A, PPARγ: Peroxisome proliferator-activated γ-receptors, eNOS: Endothelial nitric oxide synthase, MTNR1B: Melatonin receptor 1B, KCTD15: Potassium channel tetramerization domain containing 15, ADRB2: Adrenoceptor beta2, and DAT-1: dopamine transporter.
